# Antithrombotic Preventive Medication Prescription Redemption and Socioeconomic Status in Hungary in 2016: A Cross-Sectional Study

**DOI:** 10.3390/ijerph17186855

**Published:** 2020-09-19

**Authors:** Attila Juhász, Csilla Nagy, Orsolya Varga, Klára Boruzs, Mária Csernoch, Zoltán Szabó, Róza Ádány

**Affiliations:** 1 Public Health Administration Service of Government Office of Capital City Budapest, 1056 Budapest, Hungary; juhasz.attila@kmr.antsz.hu (A.J.); nagy.csilla@kmr.antsz.hu (C.N.); 2 Department of Public Health and Epidemiology, Faculty of Medicine, University of Debrecen, 4032 Debrecen, Hungary; varga.orsolya@med.unideb.hu; 3 Department of Health Systems Management and Quality Management in Health Care, Faculty of Public Health, University of Debrecen, 4032 Debrecen, Hungary; boruzs.klara@gmail.com; 4 Department of Computer Science and Library and Information Science, Faculty of Informatics, University of Debrecen, 4032 Debrecen, Hungary; csernoch.maria@inf.unideb.hu; 5 Department of Emergency Medicine, Faculty of Medicine, University of Debrecen, 4032 Debrecen, Hungary; szabo.zoltan@med.unideb.hu; 6 MTA-DE-Public Health Research Group, Department of Public Health and Epidemiology, Faculty of Medicine, University of Debrecen, 4032 Debrecen, Hungary

**Keywords:** deprivation, socioeconomic status, preventive medication, drug prescription, redemption rate, cardiovascular disease burden, primary care, antithrombotic agents, health inequalities

## Abstract

This work was designed to investigate antithrombotic drug utilization and its link with the socioeconomic characteristics of specific population groups in Hungary by a comparative analysis of data for prescriptions by general practitioners and the redeemed prescriptions for antithrombotic drugs. Risk analysis capabilities were applied to estimate the relationships between socioeconomic status, which was characterized by quintiles of a multidimensional composite indicator (deprivation index), and mortality due to thromboembolic diseases as well as antithrombotic medications for the year 2016 at the district level in Hungary. According to our findings, although deprivation is a significant determinant of mortality due to thromboembolic diseases, clusters can be identified that represent exemptions to this rule: an eastern part of Hungary, consisting of two highly deprived counties, had significantly lower mortality than the country average; by contrast, the least-deprived northwestern part of the country, consisting of five counties, had significantly higher mortality than the country average. The fact that low socioeconomic status in general and poor adherence to antithrombotic drugs irrespective of socioeconomic status were associated with increased mortality indicates the importance of more efficient control of preventive medication and access to healthcare in all districts of the country to reduce mortality due to thromboembolic diseases.

## 1. Introduction

The latest joint publication of the Organisation for Economic Co-operation and Development (OECD) and the European Commission [1] on health and access to health services illustrates the existing notable health inequalities between and within European Union (EU) member states. There are persisting inequalities in life expectancy between sexes and among groups with different socioeconomic statuses (SESs), defined mainly by education, income levels, or occupations. As shown in a Europe-wide analysis [2], “in Central and Eastern European countries inequalities in mortality have disastrously exploded since the early 1990s”. The most significant cause of mortality differences is cardiovascular disease (CVD) [3], and studies have shown that in almost all European countries, as well as in countries from other regions [4], those with a lower SES have higher rates of CVD mortality and morbidity [5]. The relative risk of premature mortality caused by CVDs is especially unfavorable in post-communist member states of the EU [6]. According to the latest available data, the years of life lost (YLLs) due to CVDs are greater than or almost equal to 10,000 YLLs per 100,000 among males in five EU member states, including Hungary, Romania, Latvia, Lithuania, and Bulgaria [7]. Although it has been shown that a remarkable decline in CVD mortality over the last 25 years was experienced in lower socioeconomic groups, “further reducing inequalities, especially in the Nordic, Central-European and Baltic countries, remains an important challenge for European health systems and policies” [8]. A recent descriptive study [9] on inequalities and premature mortality underlined that although CVD mortality in harmony with a longer-term trend in the United Kingdom (UK), which is classified as highly developed, has been decreased radically during the study period (2003–2018), there are groups, e.g., women living in deprived areas, requiring further attention. Although CVDs cover a broad range of diseases, including thromboembolic diseases, such an inverse relationship between SES and morbidity/mortality seems to present for thromboembolic diseases. For example, a nationwide study found that higher neighborhood SES was associated with a lower incidence of venous thromboembolism in the Netherlands [10]. Another nationwide study carried out in Sweden reported that high educational level and certain occupations requiring high levels of education were negatively associated with venous thromboembolism [11].

It is generally accepted that a substantial benefit can be derived from preventive medication in both primary and secondary prevention in addition to lifestyle-modifying interventions. In primary prevention, aspirin and lipid-lowering and antihypertensive drugs, as indicated by the existing symptoms, are widely used [12,13,14,15]. For secondary prevention of certain CVDs—in addition to medicines also used in primary prevention—antiplatelet drugs and anticoagulants, which exhibit their effect by blocking the pathways for the activation of platelet aggregation and the coagulation cascade, are strongly recommended. Clinical trials have demonstrated the safety and efficacy of antiplatelet medication for non-cardioembolic stroke prevention, while anticoagulants are recognized for preventing cardioembolic stroke, atrial fibrillation, and thrombotic complications related to different heart diseases [16]. The persistent long-term use of these preventive drugs has become widespread [17], and guidelines state at what levels of risk preventive drugs are recommended [1,18].

The Prospective Urban Rural Epidemiology (PURE) study illustrated that within groups of countries categorized by income (low, lower-middle, upper-middle, and high), the average use of treatment drug—among them, antiplatelet medication—for secondary prevention of CVD is low, particularly in low-income countries. Later, a study based on the results acquired in the PURE study revealed marked cross-country differences [19]. Although data on the level and availability of secondary prevention at the country level would be essential for planning and targeting national health system policies that can decrease premature CVD mortality and morbidity, only a few studies have been published on the link between the utilization of antiplatelet drugs and anticoagulants (hereafter antithrombotic agents) for preventive purposes and socioeconomic status.

A study from China demonstrated a more than 70% lower use of antiplatelet agents among patients with lower SES, while another study from Sweden showed that the odds of prescribing warfarin (common vitamin K antagonist oral anticoagulant agent) were more prominent among men and women living in high-SES neighborhoods than among their counterparts residing in low SES neighborhoods—men: odds ratio (OR) = 1.44 (95% confidence interval (CI) 1.27–1.63); women: OR = 1.19 (95% CI 1.05–1.36). Individuals in low-SES neighborhoods more often undergo inadequate treatments and less warfarin according to treatment recommendations for atrial fibrillation and likely for other cardiovascular diseases as well [20]. Contrary to a recent study based on an electronic cohort of individuals aged over 20 in Wales (UK) and using linked data from primary and secondary care followed for six years (2004–2010), no significant evidence of socioeconomic inequality was demonstrated across quintiles of the Welsh Index of Multiple Deprivation, an area-based measure of socioeconomic inequality, in the adherence to recommended medication for primary and secondary prevention of coronary heart disease [21,22].

Since physicians in general practice play a major role in initiating, coordinating, and providing long-term follow-up for the prevention of non-communicable diseases [23], our study examined the prescription and redemption rates of the most common oral antiplatelet drugs and anticoagulants prescribed for thrombosis prevention from all general practices and defined their relationships with socioeconomic status in Hungary.

Our cross-sectional ecological study aimed to examine whether inequalities exist in preventive antiplatelet and anticoagulant treatments in association with socioeconomic conditions. In the analysis, the relationships between the following were investigated:

1. Deprivation and mortality caused by thrombotic diseases;
2. Deprivation and the prescription/redemption of selected oral antiplatelet and anticoagulant drugs, with special focus on the comparison between areas with high mortality, although socioeconomically well developed and areas with relatively favorable mortality although socioeconomically deprived, and
3. The level of deprivation and the prescribing pattern of antithrombotic drugs.

## 2. Materials and Methods

In line with our previous studies on preventive medication, the methodology developed by our group [24,25,26] was used in this study.

### 2.1. Data

For 2016, mortality caused by stroke (ICD-10 I63, I64), arterial embolism and thrombosis (ICD-10 I74), phlebitis and thrombophlebitis (ICD-10 I80), portal vein thrombosis (ICD-10 I81), and other venous embolism and thrombosis (ICD-10 I82) data were acquired from the Hungarian Central Statistical Office (HCSO), while population data were obtained from the Central Office for Administrative and Electronic Public Services. Both mortality and population data for the 40+ age group used were stratified by the districts and 5-year age bands and sex.

Data on prescriptions by general practitioners (GPs) and redeemed prescriptions for the four most commonly used oral antithrombotic drugs were comparatively analyzed in Hungary during the last year of data availability (2016) for validated databases allowing district-level analysis.

The number of prescriptions and the number of redeemed prescriptions for specified oral antithrombotic drugs as prime examples of commonly used classes of antithrombotic agents for secondary cardiovascular prevention [16], such as vitamin K antagonists (a coumarin derivate, acenocoumarol—Syncumar;—all warfarin products available in Hungary), a Factor Xa inhibitor (rivaroxaban—Xarelto), and a platelet aggregation inhibitor (clopidogrel—all originator and generic products available in Hungary), were collected from the National Health Insurance Fund Administration of Hungary for each primary-healthcare practice for the entire year of 2016. According to the Hungarian regulations, general practitioners can prescribe only one type of drug as a 1-month dose of one prescription for patients with long-term medication use.

### 2.2. Deprivation Index Calculation

A deprivation index (DI) to characterize the socioeconomic deprivation at the municipality level in comparison with the national average could be calculated by using the last census data originally obtained from HCSO (Census 2011) and the Hungarian Tax and Financial Control Administration (2011), and presently available from the Regional Informational System of the Ministry of Local Government and Regional Development.

The method to calculate DI values and their efficacies to identify SES-related inequalities in CVD mortality have been explained previously [26] and has been used to conduct former studies to describe the association between deprivation and mortality amenable to healthcare [27], premature mortality attribute to alcoholic liver disease [28], statin utilization [24], between deprivation and the incidence and survival of childhood leukemia [29], as well as between deprivation and antihypertensive medication use in Hungary [25].

Briefly, the DI is based on seven municipality-level basic socioeconomic indicators, including income, level of education, rate of unemployment, proportion of one-parent families, proportion of large families, density of housing, and car ownership [26]. After natural log transformation and standardization of these variables, the area-specific indices as weighted sums of the z-scores were defined by applying a principal component analysis. Positive index values indicate districts/municipalities with a lower socioeconomic status compared with the national average, and the converse is true for districts with negative index values.

### 2.3. Study at the District Level

Hungary is divided administratively into 19 counties, in addition to the capital Budapest. Thus, it has 20 European regions at the third level of the Nomenclature of Territorial Units for Statistics (NUTS). The counties are further subdivided into 198 districts constituting Local Administrative Unit 1 (LAU1), formerly known as NUTS level 4 of Hungary [30].

The number of GP practices in Hungary serving 2809 municipalities of the country was 6199 in 2016. The number of clients registered by the GPs varied extensively (800–3000 persons/practice), and the average size was 1581 persons/practice. Generally, more family practices are available in urban areas, whereas one family practitioner provides services for more than one municipality in rural areas. Free choice of a physician at the primary care level is a norm in Hungary, and DI is not used at the practice level. Thus, to reduce the risk of misclassification, all data were aggregated to the district level. To define the frequency of prescription and that of redemption, the denominator was the size of the 40±-year-old population adjusted by sex and age. All districts included in the analysis were classified into 5 groups (quintiles), ranging from the least deprived (quintile I) to the most deprived (quintile V), with each containing one-fifth of the districts analyzed.

Applying the “disease mapping” option within the Rapid Inquiry Facility (RIF) [31], the spatial pattern of hierarchical Bayesian indirectly smoothed standardized mortality caused by stroke (ICD-10 I63, I64), arterial embolism and thrombosis (ICD-10 I74), phlebitis and thrombophlebitis (ICD-10 I80), portal vein thrombosis (ICD-10 I81), and other venous embolism and thrombosis (ICD-10 I82) for the 40+ age group for 2016. It was examined and visualized with posterior probabilities at the district level [32,33]. The frequency of prescriptions for the selected oral antithrombotic agents, redeemed prescriptions, and the ratios for compliance in relation to the national average was also mapped using the RIF, and their association with deprivation was defined using quintiles of DI as a district-based categorical covariate.

### 2.4. Statistics

The ratios between the number of redeemed prescriptions and that of the prescriptions for antithrombotic drugs were utilized to define the characteristics of the level of primary non-compliance. The results were analyzed by types of drugs and deprivation quintiles.

## 3. Results

### 3.1. The Spatial Distribution of Mortality Caused by Different Thromboembolic Diseases in Connection with Deprivation

**Figure 1:**
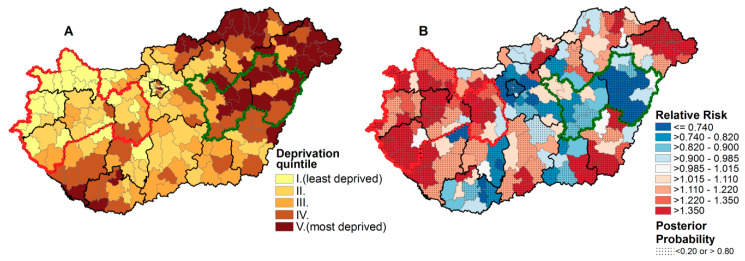
The spatial distribution of deprivation (**A**) and mortality risk in the age group 40+ years due to thromboembolic diseases (ICD-10.: I63-I64, I74, I80-I82) (**B**), Hungary, 2016. A cluster of counties least deprived with a high mortality rate is bordered by a red line, while another cluster of counties highly deprived with low mortality rate is bordered with a green line.

As was described in our previous publications [24,25] and also demonstrated in the present paper, deprivation index values defined by districts varied from −3.76 to +5.83, as shown in Figure 1A, which indicated a high level of socioeconomic inequalities in the country. The quintiles based on the DI values were defined as ranging from −3.76 ≤ DI ≤ −1.13, with an average of −1.72 (quintile I); −1.13 < DI ≤ −0.43, with an average of −0.78 (quintile II); −0.43 < DI ≤ 0.22, with an average of −0.12 (quintile III); 0.22 < DI ≤ 1.06, with an average of 0.62 (quintile IV); and 1.06 < DI ≤ 5.83, with an average of 1.97 (quintile V). The distribution of the DI values indicated that the least-favored districts were detected in the northeastern and southwestern parts of Hungary in 2011, the year of the last census. The least-deprived districts of the country were localized at the northwestern part of Hungary, in the capital city of Budapest, and its neighboring areas. The spatial distribution of mortality due to thromboembolic diseases could be characterized by significant inequalities, and the areas of highest standardized mortality ratios (SMRs) were detected in the northwestern, western, northeastern, and southeastern regions of the country. However, a cluster consisting of five counties with high mortality caused by thromboembolic diseases was detected in the northwestern and western parts of the country (circle drawn by the dashed red line), where the majority of the least-deprived districts were localized, while a cluster consisting of two counties (circle drawn by the dashed green line) that were highly deprived with low mortality figures was localized in the northeastern region (Figure 1A,B).

The results of the regression analysis presented that deprivation was a significant but non-linear determinant of mortality due to thromboembolic diseases in deprivation quintiles III–IV, and it was found to be higher by approximately 30–40%, while in quintile V, it was approximately 30% higher than that in the least-deprived region (quintile I) (Table 1).

### 3.2. Prescription and Redemption Rates of Antithrombotic Drugs and Their Relationships with Deprivation

In Hungary, 1,729,058 clopidogrel, 1,024,233 Syncumar, 447,050 warfarin, and 165,816 Xarelto prescriptions for 28 days/one month were issued by general practitioners in 2016. The highest frequencies of both prescription and redemption per person aged 40+ years old were noticed for clopidogrel, and the lowest frequencies were shown for Xarelto. The redemption rate of the four types of antithrombotic drugs differed significantly. The redemption rate of Syncumar prescriptions was the highest, 76.24%, while the redemption rate of clopidogrel was the lowest, only with 54.4% (Table 2).

**Figure 2:**
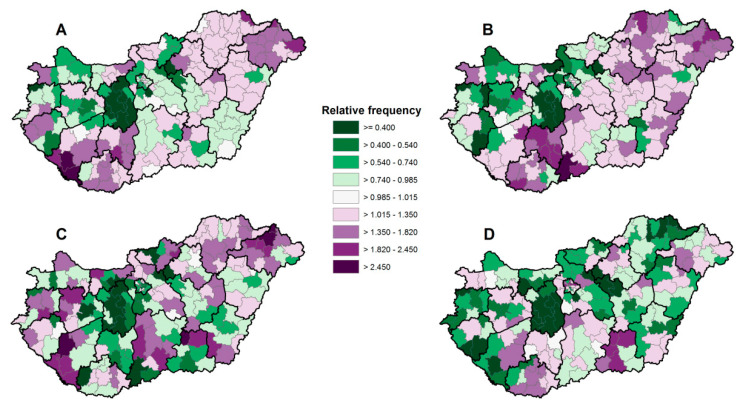
The spatial distribution of the relative frequencies of Clopidogrel (**A**), Syncumar (**B**), Warfarin (**C**), and Xarelto (**D**) prescription at the district level, Hungary, 2016.

The frequency of clopidogrel prescriptions in relation to the national average was higher in districts in the southwestern and northeastern parts of Hungary. The frequency of Syncumar prescriptions also had a similar spatial pattern with a higher relative prescription rate in the middle of the country (Figure 2A,B). The districts with a higher relative frequency of warfarin prescriptions were located in the northeastern, southern, and western parts of Hungary (Figure 2C). Districts with a higher relative prescription ratio for Xarelto were located along the axis in the southwestern and southeastern parts of the country (Figure 2D).

**Figure 3:**
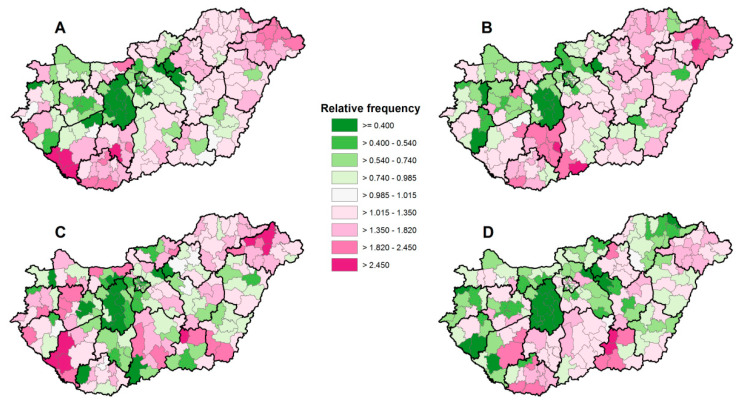
The spatial distribution of the relative frequencies of Clopidogrel (**A**), Syncumar (**B**), Warfarin (**C**), and Xarelto (**D**) redemption at the district level, Hungary, 2016.

The spatial patterns of relative redemption ratio were very similar to prescription ratio for each anticoagulant drug, respectively (Figure 3A–D.)

### 3.3. Prescription and Redemption Patterns of Antithrombotic Drugs and Their Relationships with Deprivation Levels

**Figure 4:**
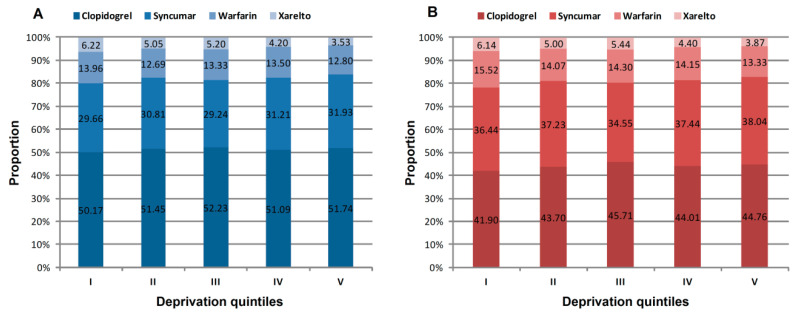
Antithrombotic drug prescription (**A**) and redemption (**B**) by deprivation index (DI) quintile and drug types, Hungary, 2016.

Concerning the total number of drugs utilized, clopidogrel prescription and redemption were the highest (approximately half of the total prescriptions and 40.7% of the total redemptions), followed by the prescription and redemption of Syncumar, warfarin, and Xarelto. The proportions of clopidogrel and Syncumar prescriptions and redemptions were increased by deprivation level, while the proportion of warfarin and Xarelto prescriptions and redemptions showed an opposite trend, i.e., in the most-deprived quintile, the proportions were much lower than in the least-deprived quintile. The most remarkable differences between the least-deprived and the most-deprived quintiles were observed for Xarelto prescription and redemption (6.22% vs. 3.53% and 6.14% vs. 3.87%, respectively) (Figure 4).

A significant association was discovered between the frequency of clopidogrel prescriptions per 100 persons aged 40+ years and deprivation. The incidence rate ratios in the highest deprivation quintile (quintile V) were 60.7% higher than those in the lowest quintile area (quintile I). A similar association was found in the case of Syncumar prescriptions, with 25.342 prescriptions/100 persons in the most-deprived quintile (Table 3). The results show that deprivation is a significant but non-linear determinant of the frequency of warfarin prescriptions, and no association was found between Xarelto prescription frequency and deprivation. Similar but stronger, positive associations were detected between deprivation and redemption regarding clopidogrel, Syncumar, and warfarin, but no association was found for Xarelto.

When the number of redeemed prescriptions and that of the prescriptions for antithrombotic drugs were compared, the frequency of redemption and the redemption rate were shown to be increased as the deprivation became more pronounced, i.e., better primary compliance was observed in quintile V than in quintile I, and the highest rates (independent of the types of drugs) were always associated with the districts with the highest deprivation (Table 3).

### 3.4. Identification of Anomalies in Preventive Medication in a Cluster with High Mortality in the Less Deprived Region of the Country

The cluster with high mortality (standardized mortality ratio: 1.393) caused by thromboembolic diseases in the northwestern part of the country, consisting mainly of counties with the highest socioeconomic performance, was further analyzed, and it was shown that both prescription and redemption rates for each drug were less favorable than the country average (Table 4). To better demonstrate the extent of the discrepancy between socioeconomic development and thromboembolic disease mortality, as well as preventive medication, further analysis was performed. Prescription and redemption rates, as well as the relative redemption rate, were defined for each drug for an eastern Hungarian region consisting of two highly deprived counties with significantly lower mortality than the country average (standardized mortality ratio: 0.747, and all the rates were found to be significantly higher than that in the northwestern cluster, close to or even better than the national average values).

## 4. Discussion

In our previous studies, a notable positive association between the relative risk of premature cardiovascular mortality and deprivation was shown in Hungary [24,25,26]. Regarding preventive medication at the primary-care level, high inequalities linked with socioeconomic deprivation were shown both in the case of lipid-lowering treatment (statin medication) and of antihypertensive medication [24,25]. In the country-wide analyses of statin and antihypertensive medication, mapping the frequency of prescriptions, redeemed prescriptions, and ratios for primary compliance in comparison to the national average was done, and were defined by their associations with deprivation (with tertiles of the deprivation index as a district-based categorical covariate) [24]. In the case of statin utilization, a low relative frequency of statin prescriptions was noticed in districts with the highest deprivation; however, significantly higher primary compliance (redemption) was noted in these districts. Data from this study suggested that lack of statin utilization may illustrate a significant barrier to reducing CVD mortality, particularly among people living in highly deprived areas of the country. Similarly, risk analysis of antihypertensive medication [25] showed a significant association between premature cardiovascular and cerebrovascular mortality risk and deprivation. The patterns of antihypertensive drug prescription and redemption significantly differed by DI tertile. In areas with the highest deprivation, higher relative frequencies of angiotensin-converting enzyme inhibitors, beta-blockers, and calcium channel blocker prescriptions and lower relative frequency of angiotensin II receptor blocker prescriptions were found. The proportion of angiotensin II receptor blockers among the antihypertensive medications used elevated with the improvement in socioeconomic status.

Our current study was designed to examine antithrombotic drug utilization and its relationship with the socioeconomic characteristics of different population groups in Hungary with a focus on the comparative analysis of data for prescriptions by general practitioners and the redeemed prescriptions for antithrombotic drugs.

Among the four preventive drugs, the redemption rate of Syncumar prescriptions was the highest, 76.24%, while the redemption rate of clopidogrel was the lowest, only 54.4%. The frequency of prescription for these drugs (except for Xarelto) was positively associated with deprivation. In fact, as the deprivation became more pronounced, the redemption rate was noticed to increase, irrespective of the types of drugs. However, the interactions between prescription/redemption measures and mortality due to thromboembolic diseases among districts were not consequent in Hungary. In a cluster of high mortality caused by thromboembolic diseases in the northwestern part of the country, consisting mainly of counties with the highest socioeconomic performance, prescription and redemption rates for each drug were found to be less favorable than the country average. In contrast, an eastern Hungarian region consisting of two highly deprived counties with significantly lower mortality than the country average had a significantly higher prescription and redemption rates than this northwestern cluster. The reason why these discrepancies exist cannot be squarely defined. It is reasonable to suppose that a study on healthcare access and performance would be able to—at least partly—clarify the background of these unexpected inequalities. There are 36 stroke units in Hungary, of which performance is assessed by the thrombolysis rate (national thrombolysis rate is 13%). Only a single stroke unit (Veszprém) in the northwestern cluster slightly overperformed (14%) compared to the average, lagging behind the performance of centers in Budapest and Debrecen responsible for the healthcare of the population of the eastern cluster with significantly better mortality rates of thromboembolic diseases and better coverage in preventive medication. Such deficiency in healthcare performance might be explained by human resource deficits in the northwestern cluster caused by increased out-migration and, consequently, the attrition of physicians after the EU accession of Hungary [34]. In addition, a significant portion of physicians living in the northwestern part of the country are cross-border commuters working in Austrian healthcare institutions [35], especially in the bordering province of Burgenland, where, in certain hospitals, the proportion of commuting Hungarian doctors reaches 23%. This fact became known due to the connection with the COVID-19 pandemic [36].

In line with our findings, insufficient anticoagulant treatment and its link with SES have been reported by several studies [37]. Prescribing was less frequent with older age and in patients born in other Nordic countries or countries outside of Europe than in those born in Sweden. University education and higher income were associated with higher levels of oral anticoagulant prescription [38].

In an Italian study based on the Italian geographical macro-regions (North, Central, South), data on medication adherence of nonvalvular atrial fibrillation patients were analyzed to assess whether socioeconomic conditions might also influence medication adherence. Regional disparities exist in drug prescriptions. In high-risk patients, oral anticoagulants were more likely to be prescribed in northern and central patients than in high-risk southern patients. Additionally, medication adherence was noticed as a progressive decrease from North to South [39].

A review presented 47 articles concerning anticoagulant therapy on patients’ perspectives and patients’ adherence. The findings through five interacting dimensions of adherence were synthesized: patient-related factors, therapy-related factors, condition-related factors, health system factors, and social, economic factors [40]. Overall, one-fifth of the studies in the review examined the impact of patients’ background in social and economic aspects on their perception of and adherence to warfarin therapy [41,42,43,44]. The financial burden of warfarin to patients is dependent on healthcare service costs to individuals and the extent to which these medicines are subsidized by the governments or insurance companies in those countries where they are available. In regard to warfarin, only two studies stated that a small number of patients were financially burdened by further expenses associated with managing the therapy [41,44].

In Hungary, the higher redemption rate of the antithrombotics—including the high-cost Xarelto—in deprived districts might be explained by the broad access to prescription exemption certificates. In contrast, the vitamin K antagonist warfarin applied in acute thromboembolic diseases as anticoagulant therapy could cause similar redemption patterns across quintiles.

The effectiveness of oral anticoagulants is critically dependent on patients’ adherence to intake regimens. Lee et al. underlined the significant role of promoting medication adherence for risk reduction, especially in low-income patients with CVDs [45].

### 4.1. Implications

Contrary to the approach of “the lower social, economic status associated with lower adherence” [46], our study indicates that a more complex relationship exists between socioeconomic factors and medication adherence. There is a need to address medication nonadherence, which is an important cost driver in healthcare expenditures and inequalities [47], as an interconnected network. Beyond policies targeting health behavior (new taxes on “unhealthy” food, such as sweetened, salty, and fatty products, anti-smoking legislation) that were introduced by the Hungarian government, actions to facilitate favorable changes in inequalities in health services are necessary. For example, financial incentives are effective tools to achieve a major contribution to the reduction in inequalities in the delivery of clinical care related to area deprivation [48]. Additionally, it can be supposed that underlying problems exist at the level of service provision and/or access to health services in certain clusters given the fact that low prescription and redemption rates occur together with high SES. In general, an analysis of the spatial distribution pattern of mortality in connection with that of the relevant preventive medication can result in the identification of regions/populations with no adequate treatment, including preventative medication, which may help to identify gaps in service provision and to diminish preventable and amenable mortality.

### 4.2. Strengths and Weaknesses of the Study

Only limited literature is available on the relationship between antithrombotic drug utilization and SES. Additionally, the majority is focused on warfarin. To the best of our knowledge, this is the first comprehensive study that analyzed the relationship of SES to mortality caused by thromboembolic diseases and the utilization of antithrombotic drugs in a post-communist member state of the EU, which is characterized by an extremely high relative risk of premature CVD mortality by applying a multidimensional composite indicator to properly characterize the SES and using risk analysis capabilities to estimate the relationships between deprivation and thromboembolic disease burden as well as antithrombotic medication. An additional strength of this study is the complete coverage of the entire population. However, some limitations need to be considered in the interpretation of our findings. In our observational study with the methodology of cross-sectional and ecological studies, the statistical associations detected do not indicate a causal relationship, and the relationships observed between deprivation and mortality, as well as deprivation and preventive medication at the population level, may not be linked with mortality and preventive medication in individuals [49]. There are factors (e.g., related to patients, physicians, and health system) with an actual impact on antithrombotic drug utilization not included in the study [50]. In Hungary, the high prevalence of health behavior risk factors (such as smoking, uncontrolled alcohol consumption, physical inactivity, unhealthy nutrition) is a severe problem regarding the prevention of chronic diseases [51], and these factors were unmeasured confounders in our study.

## 5. Conclusions

The combined effect of deprivation and adherence to preventive medication on cardiovascular mortality was addressed by this work. Although deprivation is a significant determinant of mortality due to thromboembolic diseases, the identified clusters underlined that a complex relationship exists between socioeconomic factors and medication adherence. A linear association was noticed between the relative frequency of prescriptions/redemptions and deprivation for most antithrombotic drugs, except Xarelto, and the incidence rate ratio of redemption and redemption rate was found to be higher as deprivation became more pronounced. However, prescription and redemption rates of the severely deprived eastern Hungarian counties were at the level of the country average, while in the least-deprived northwestern cluster, these rates were significantly lower. Our analysis of the spatial distribution pattern of mortality in connection with that of the relevant preventive medication can result in the identification of regions/populations with no adequate treatment, thus in more effective service provision to diminish preventable and amenable mortality.

## Figures and Tables

**Table 1:** Mortality risk due to thromboembolic diseases for 40–X age group of the population at the district level by deprivation index (DI) quintiles, Hungary, 2016.

DI Quintiles	Observed Cases	Incidence Rate Ratio (95%CI)
I. (least deprived)	1093	1
II.	1306	1.229 (1.025–1.473)
III.	1378	1.284 (1.070–1.541)
IV.	1070	1.387 (1.154–1.666)
V. (most deprived)	735	1.304 (1.081–1.574)
*p*-trend		0.0067

**Table 2:** Anticoagulant utilization (prescription, redemption, and redemption rate) by drug types, Hungary, 2016.

Drug Types	Total Number of Prescriptions	Total Number of Redemptions	Frequency of Prescription	Frequency of Redemption	Redemption Rate (%)
	(Per 100 Persons Aged 40+ Years)				
Clopidogrel	1,729,058	941,921	32.322 (32.290–32.355)	17.608 (17.578–17.638)	54.476 (54.402–54.550)
Syncumar	1,024,233	780,918	19.147 (19.118–19.176)	14.598 (14.571–14.625)	76.244 (76.162–76.326)
Warfarin	447,050	305,034	8.357 (8.334–8.380)	5.702 (5.0–5.721)	68.232 (68.096–68.369)
Xarelto	165,816	107,069	3.099 (3.085–3.114)	2.002 (1.990–2.013)	64.571 (64.34–64.801)

**Table 3:** Frequency of prescription and redemption of oral antithrombotic drugs per 100 persons aged 40+ years by DI quintiles at the district level, Hungary, 2016.

Drug Types	DI Quintiles					*p*-Trend
	I. (Least Deprived)	II.	III.	IV.	V. (Most Deprived)	
	Prescription					
	Frequency (Per 100 Persons Aged 40+ Years) (95% CI)					
Clopidogrel	26.373 (26.308–26.438)	29.378 (29.308–29.448)	34.007 (33.941–34.072)	35.900 (35.827–35.986)	40.654 (40.562–40.746)	<0.0001
Syncumar	15.51 (15.454–15.565)	17.546 (17.485–17.606)	19.061 (19.003–19.120)	22 (21.928–22.073)	25.342 (25.254–25.431)	<0.0001
Warfarin	7.328 (7.285–7.372)	7.246 (7.201–7.292)	8.691 (8.644–8.738)	9.5 (9.443–9.558)	10.051 (9.980–10.122)	<0.0001
Xarelto	3.255 (3.225–3.285)	2.876 (2.847–2.906)	3.387 (3.357–3.418)	2.96 (2.926–2.994)	2.795 (2.756–2.835)	<0.0001
**Drug Types**	**Redemption**					***p*-Trend**
	**Frequency (per 100 Persons Aged 40+ Years) (95% CI)**					
Clopidogrel	12.774 (12.719–12.828)	15.750 (15.688–15.812)	18.897 (18.835–18.959)	20.118 (20.042–20.193)	24.166 (24.072–24.261)	<0.0001
Syncumar	11.054 (11.004–11.104)	13.378 (13.322–13.434)	14.294 (14.240–14.349)	17.169 (17.100–17.238)	20.791 (20.705–20.878)	<0.0001
Warfarin	4.724 (4.688–4.760)	5.066 (5.028–5.105)	5.918 (5.879–5.958)	6.477 (6.429–6.527)	7.219 (7.158–7.281)	<0.0001
Xarelto	1.866 1.843–1.889]	1.796 (1.772–1.820)	2.249 (2.224–2.274)	2.017 (1.989–2.046)	2.111 (2.076–2.146)	<0.0001
**Drug Types**	**Redemption Rate (%) (95% CI)**					***p*-Trend**
Clopidogrel	48.599 (48.43–48.769)	53.622 (53.457–53.788)	55.572 (55.427–55.718]	55.952 (55.783–56.121)	59.18 (58.992–59.368)	<0.0001
Syncumar	71.258 (71.059–71.458)	76.224 (76.041–76.407)	75.013 (74.844–75.183]	78.033 (77.853–78.214)	82.008 (81.821–82.196)	<0.0001
Warfarin	64.402 (64.095–64.71)	69.851 (69.543–70.159)	68.093 (67.823–68.363]	68.268 (67.961–68.577)	72.025 (71.68–72.372)	<0.0001
Xarelto	57.279 (56.805–57.756)	62.418 (61.905–62.935)	66.357 (65.919–66.796]	68.246 (67.696–68.8)	75.63 (75.004–76.26)	<0.0001

**Table 4:** Mortality rate of the population aged 40+ years to thromboembolic diseases and frequency of the prescription and redemption, as well as redemption rate of oral antithrombotic drugs in the less deprived counties with high mortality (bordered by a red line on Figure 1) of the northwestern part of Hungary and in highly deprived counties with a low mortality rate (bordered by a green line on Figure 1) of the eastern part of Hungary in comparison with the national average data, 2016.

	Less Deprived Counties with High Mortality				Highly Deprived Counties with Low Mortality Rate				Hungarian National Average			
Standardized * Death Rate (Per 100,000 Persons Aged 40+ Years) (95% CI)	92.097 (87.140–97.282)				49.728 [44.749–55.121]				66.493 [64.645–68.394]			
	Clopidogrel	Syncumar	Warfarin	Xarelto	Clopidogrel	Syncumar	Warfarin	Xarelto	Clopidogrel	Syncumar	Warfarin	Xarelto
**Prescription frequency (per 100 persons aged 40+ years) [95% CI]**	23.769 (23.684–23.832)	13.102 (13.035–13.161)	7.537 (7.485–7.582)	2.146 (2.119–2.171)	30.867 (30.737–30.966)	20.234 (20.122–20.335)	7.577 (7.503–7.639)	2.664 (2.619–2.703)	32.322 (32.290–32.355)	19.147 (19.118–19.176)	8.357 (8.334–8.380)	3.099 (3.085–3.114)
**Redemption frequency (per 100 persons aged 40+ years) [95% CI]**	12.857 (12.790–12.925)	9.965 (9.905–10.017)	5.320 (5.275–5.349)	1.369 (1.346–1.369)	18.026 (17.918–18.14)	16.025 (15.922–16.096)	5.125 (5.063–5.17)	1.863 (1.825–1.864)	17.608 (17.578–17.638)	14.598 (14.571–14.625)	5.702 (5.683–5.721)	2.002 (1.990–2.013)
**Redemption rate [95% CI]**	54.09 (53.887–54.271)	76.056 (75.819–76.227)	70.584 (70.252–70.751)	63.742 (63.085–63.749)	58.398 (58.148–58.632)	79.196 (78.941–79.338)	67.631 (67.152–67.917)	69.926 (69.131–69.935)	54.476 (54.402–54.550)	76.244 (76.162–76.326)	68.232 (68.096–68.369)	64.571 (64.34–64.801)

* Standard population: 2013 European Standard Population.

## References

[1] OECD/European Union (2018). Health at a Glance: Europe 2018: State of Health in the EU Cycle.

[2] Mackenbach J.P. (2017). Nordic paradox, southern miracle, eastern disaster: Persistence of inequalities in mortality in Europe. Eur. J. Public Health.

[3] World Health Organization Global Health Estimates 2016: Disease Burden by Cause, Age, Sex, by Country and by Region, 2000–2016. https://www.who.int/healthinfo/global_burden_disease/estimates/en/index1.html.

[4] Roth G.A., Johnson C., Abajobir A., Abd-Allah F., Abera S.F., Abyu G., Ahmed M., Aksut B., Alam T., Alam K. (2017). Global, regional, and national burden of cardiovascular diseases for 10 causes, 1990 to 2015. J. Am. Coll. Cardiol..

[5] Mackenbach J.P., Cavelaars A.E.J.M., Kunst A.E., Groenhof F., Andersen O., Borgan J.K., Costa G., Crialesi R., Desplanques G., Faggiano F. (2000). Socioeconomic inequalities in cardiovascular disease mortality. An international study. Eur. Heart J..

[6] Karanikolos M., Adany R., McKee M. (2017). The epidemiological transition in Eastern and Western Europe: A historic natural experiment. Eur. J. Public Health.

[7] Institute for Health Metrics and Evaluation (IHME) GBD Compare. https://vizhub.healthdata.org/gbd-compare/.

[8] Di Girolamo C., Nusselder W.J., Bopp M., Brønnum-Hansen H., Costa G., Kovács K., Leinsalu M., Martikainen P., Pacelli B., Rubio Valverde J. (2020). Progress in reducing inequalities in cardiovascular disease mortality in Europe. Heart.

[9] Lewer D., Jayatunga W., Aldridge R.W., Edge C., Marmot M., Story A., Hayward A. (2020). Premature mortality attributable to socioeconomic inequality in England between 2003 and 2018: An observational study. Lancet Public Health.

[10] Kort D., van Rein N., van der Meer F.J.M., Vermaas H.W., Wiersma N., Cannegieter S.C., Lijfering W.M. (2017). Relationship between neighborhood socioeconomic status and venous thromboembolism: Results from a population-based study. J. Thromb. Haemost..

[11] Zöller B., Li X., Sundquist J., Sundquist K. (2012). Socioeconomic and occupational risk factors for venous thromboembolism in Sweden: A nationwide epidemiological study. Thromb. Res..

[12] Ettehad D., Emdin C.A., Kiran A., Anderson S.G., Callender T., Emberson J., Chalmers J., Rodgers A., Rahimi K. (2016). Blood pressure lowering for prevention of cardiovascular disease and death: A systematic review and meta-analysis. Lancet.

[13] Arnett D.K., Blumenthal R.S., Albert M.A., Buroker A.B., Goldberger Z.D., Hahn E.J., Himmelfarb C.D., Khera A., Lloyd-Jones D., McEvoy J.W. (2019). 2019 ACC/AHA guideline on the primary prevention of cardiovascular disease: A report of the American college of cardiology/American heart association task force on clinical practice guidelines. J. Am. Coll. Cardiol..

[14] Sutcliffe P., Connock M., Gurung T., Freeman K., Johnson S., Ngianga-Bakwin K., Grove A., Gurung B., Morrow S., Stranges S. (2013). Aspirin in primary prevention of cardiovascular disease and cancer: A systematic review of the balance of evidence from reviews of randomized trials. PLoS ONE.

[15] Chou R., Dana T., Blazina I., Daeges M., Jeanne T.L. (2016). Statins for prevention of cardiovascular disease in adults: Evidence report and systematic review for the US preventive services task force. JAMA.

[16] Kapil N., Datta Y.H., Alakbarova N., Bershad E., Selim M., Liebeskind D.S., Bachour O., Rao G.H.R., Divani A.A. (2016). Antiplatelet and anticoagulant therapies for prevention of ischemic stroke. Clin. Appl. Thromb. Hemost..

[17] Patrono C., Morais J., Baigent C., Collet J.P., Fitzgerald D., Halvorsen S., Rocca B., Siegbahn A., Storey R.F., Vilahur G. (2017). Antiplatelet agents for the treatment and prevention of coronary atherothrombosis. J. Am. Coll. Cardiol..

[18] Zerah L., Bun R.-S., Guillo S., Collet J.-P., Bonnet-Zamponi D., Tubach F. (2019). A prescription support-tool for chronic management of oral antithrombotic combinations in adults based on a systematic review of international guidelines. PLoS ONE.

[19] Yusuf S., Islam S., Chow C.K., Rangarajan S., Dagenais G., Diaz R., Gupta R., Kelishadi R., Iqbal R., Avezum A. (2011). Use of secondary prevention drugs for cardiovascular disease in the community in high-income, middle-income, and low-income countries (the PURE Study): A prospective epidemiological survey. Lancet.

[20] Carlsson A.C., Wändell P., Gasevic D., Sundquist J., Sundquist K. (2015). Neighborhood deprivation and warfarin, aspirin and statin prescription A cohort study of men and women treated for atrial fibrillation in Swedish primary care. Int. J. Cardiol..

[21] King W., Lacey A., White J., Farewell D., Dunstan F., Fone D. (2018). Socioeconomic inequality in medication persistence in primary and secondary prevention of coronary heart disease—A population-wide electronic cohort study. PLoS ONE.

[22] Di Nisio M., van Es N., Büller H.R. (2016). Deep vein thrombosis and pulmonary embolism. Lancet.

[23] Mauskop A., Borden W.B. (2011). Predictors of statin adherence. Curr. Cardiol. Rep..

[24] Boruzs K., Juhász A., Nagy C., Ádány R., Bíró K. (2016). Relationship between statin utilization and socioeconomic deprivation in Hungary. Front. Pharmacol..

[25] Boruzs K., Juhász A., Nagy C., Szabó Z., Jakovljevic M., Bíró K., Ádány R. (2018). High inequalities associated with socioeconomic deprivation in cardiovascular disease burden and antihypertensive medication in Hungary. Front. Pharmacol..

[26] Juhász A., Nagy C., Páldy A., Beale L. (2010). Development of a Deprivation Index and its relation to premature mortality due to diseases of the circulatory system in Hungary, 1998–2004. Soc. Sci. Med..

[27] Nagy C., Juhász A., Beale L., Páldy A. (2012). Mortality amenable to health care and its relation to socio-economic status in Hungary, 2004–2008. Eur. J. Public Health.

[28] Nagy C., Juhász A., Papp Z., Beale L. (2013). Hierarchical spatio-temporal mapping of premature mortality due to alcoholic liver disease in Hungary, 2005–2010. Eur. J. Public Health.

[29] Jakab Z., Juhasz A., Nagy C., Schuler D., Garami M. (2017). Trends and territorial inequalities of incidence and survival of childhood leukaemia and their relations to socioeconomic status in Hungary, 1971–2015. Eur. J. Cancer Prev..

[30] Eurostat (2009). European Regional and Urban Statistics Reference Guide.

[31] Beale L., Hodgson S., Abellan J.J., Lefevre S., Jarup L. (2010). Evaluation of spatial relationships between health and the environment: The rapid inquiry facility. Environ. Health Perspect..

[32] Besag J., York J., Mollié A. (1991). Bayesian image restoration, with two applications in spatial statistics. Ann. Instit. Stat. Math..

[33] Richardson S., Thomson A., Best N., Elliott P. (2004). Interpreting posterior relative risk estimates in disease-mapping studies. Environ. Health Perspect..

[34] Varga J. (2017). Out-migration and attrition of physicians and dentists before and after EU accession (2003 and 2011): The case of Hungary. Eur J. Health Econ..

[35] Perchinig B., Horváth V., Molnár D., Tavodová L. Cross-Border Mobilities in the Austrian-Hungarian and Austrian-Slovak Border Regions. www.reminder-project.eu.

[36] BVZ at Burgenland Ungarisches Spitalspersonal im Burgenland umworben. https://www.bvz.at/burgenland/chronik-gericht/nach-grenzschliessung-ungarisches-spitalspersonal-im-burgenland-umworben-burgenland-burgenland-epidemie-viruserkrankung-corona-coronavirus-196675677.

[37] Ouali S., Mechri M., Ben Ali Z., Boudiche S., Ben Halima M., Rejaibi S., Mourali M.S., Larbi N., Meghaieth F. (2019). Factors associated to adequate time in therapeutic range with oral vitamin K antagonists in Tunisia. Tunis. Med..

[38] Sjölander M., Eriksson M., Asplund K., Norrving B., Glader E.L. (2015). Socioeconomic inequalities in the prescription of oral anticoagulants in stroke patients with atrial fibrillation. Stroke.

[39] Raparelli V., Proietti M., Buttà C., Di Giosia P., Sirico D., Gobbi P., Corrao S., Davì G., Vestri A.R., Perticone F. (2014). Medication prescription and adherence disparities in non valvular atrial fibrillation patients: An Italian portrait from the ARAPACIS study. Intern. Emerg. Med..

[40] Pandya E.Y., Bajorek B. (2017). Factors affecting patients’ perception on, and adherence to, anticoagulant therapy: Anticipating the role of direct oral anticoagulants. Patient.

[41] Dantas G.C., Thompson B.V., Manson J.A., Tracy C.S., Upshur R.E.G. (2004). Patients’ perspectives on taking warfarin: Qualitative study in family practice. BMC Fam. Pract..

[42] Platt A.B., Localio A.R., Brensinger C.M., Cruess D.G., Christie J.D., Gross R., Parker C.S., Price M., Metlay J.P., Cohen A. (2008). Risk factors for nonadherence to warfarin: Results from the IN-RANGE study. Pharmacoepidemiol. Drug Saf..

[43] Orensky I.A., Holdford D.A. (2005). Predictors of noncompliance with warfarin therapy in an outpatient anticoagulation clinic. Pharmacotherapy.

[44] Choi J.C., DiBonaventura M.D., Kopenhafer L., Nelson W.W. (2014). Survey of the use of warfarin and the newer anticoagulant dabigatran in patients with atrial fibrillation. Patient Prefer. Adherence.

[45] Lee H., Park J.H., Floyd J.S., Park S., Kim H.C. (2019). Combined effect of income and medication adherence on mortality in newly treated hypertension: Nationwide study of 16 million person-years. J. Am. Heart Assoc..

[46] Ferdinand K.C., Senatore F.F., Clayton-Jeter H., Cryer D.R., Lewin J.C., Nasser S.A., Fiuzat M., Califf R.M. (2017). Improving medication adherence in cardiometabolic disease: Practical and regulatory implications. J. Am. Coll. Cardiol..

[47] Simpson S.H., Eurich D.T., Majumdar S.R., Padwal R.S., Tsuyuki R.T., Varney J., Johnson J.A. (2006). A meta-analysis of the association between adherence to drug therapy and mortality. BMJ.

[48] Doran T., Fullwood C., Kontopantelis E., Reeves D. (2008). Effect of financial incentives on inequalities in the delivery of primary clinical care in England: Analysis of clinical activity indicators for the quality and outcomes framework. Lancet.

[49] Morgenstern H. (1995). Ecologic studies in epidemiology: Concepts, principles, and methods. Annu. Rev. Public Health.

[50] Radevic S., Kocic S., Jakovljevic M. (2016). Self-assessed health and socioeconomic inequalities in Serbia: Data from 2013 national health survey. Front. Pharmacol..

[51] Sándor J., Nagy A., Földvári A., Szabó E., Csenteri O., Vincze F., Sipos V., Kovács N., Pálinkás A., Papp M. (2017). Delivery of cardio-metabolic preventive services to Hungarian Roma of different socio-economic strata. Fam. Pract..

